# Quercetin and Its Metabolites: Mechanistic Insights as the Basis of Their Therapeutic Potential in NAFLD and HCC

**DOI:** 10.3390/molecules30224441

**Published:** 2025-11-17

**Authors:** Zhengwen Li, Yongzuo Li, Tianqing Jiang, Yue Wang, Chujie Li, Zhengyou He

**Affiliations:** 1 School of Pharmacy, Chengdu University, 2025 Chengluo Avenue, Chengdu 610106, China; liyongzuo@stu.cdu.edu.cn (Y.L.);; 2 Cell Biology-Inspired Tissue Engineering, Institute for Technology-Inspired, Regenerative Medicine, Maastricht University, 6200 MD Maastricht, The Netherlands; yue.wang@maastrichtuniversity.nl; 3 Precision Medicine, Faculty of Health, Medicine and Life Sciences, Maastricht University, 6200 MD Maastricht, The Netherlands; c.li@maastrichtuniversity.nl; 4 Laboratory of Chinese Medicine Resources and New Product Development, School of Pharmacy, Chengdu University, Chengdu 610106, China

**Keywords:** quercetin, metabolites, NAFLD, HCC, redox modulation, microbiota

## Abstract

Quercetin, a dietary flavonoid, has demonstrated antioxidant, anti-inflammatory, and anti-tumor activities. Increasing evidence highlights that its metabolites contribute to these health benefits. This review summarizes current knowledge on the molecular mechanisms and therapeutic potential of quercetin and its metabolites in non-alcoholic fatty liver disease (NAFLD) and hepatocellular carcinoma (HCC), with a focus on redox modulation and microbiota interactions. Substantial preclinical evidence supports the protective effects of quercetin and its metabolites in NAFLD and HCC. However, clinical translation is hampered by poor bioavailability, potential redox–drug interactions, and incomplete understanding of the precise molecular mechanisms involved. Future research should prioritize further elucidating the molecular mechanisms, as they represent the foundation for the rational use of quercetin and metabolite-based derivatives in the prevention and treatment of liver-related diseases. In particular, strategies that direct the activity of these bioactive compounds to their desired sites of action—by exploiting differences between normal and cancer cells—warrant more in-depth investigation.

## 1. Introduction

Quercetin is a widely studied flavonoid found in fruits, vegetables, and medicinal plants, and it has attracted significant attention due to its antioxidant, anti-inflammatory, and anti-tumor activities [1,2,3,4]. It has gained considerable attention for its potential role in disease prevention and health promotion. In recent years, particular focus has been given to its role in liver diseases, indicating that quercetin reduces hepatic steatosis and inflammation, inhibits profibrotic processes, and improves biochemical markers of liver function (e.g., ALT, AST) [5,6,7,8]. For example, a controlled clinical study reported that daily administration of 500 mg quercetin for 12 weeks lowers hepatic fat content in patients with non-alcoholic fatty liver disease (NAFLD) [9], underlining its translational potential in metabolic liver disease [10]. Currently, based on expert recommendations from 2023, the disease is referred to as Metabolic Dysfunction Associated Steatotic Liver Disease (MASLD). However, since NAFLD is more well known, we still use NAFLD in this review [11].

NAFLD commonly progresses from simple steatosis to non-alcoholic steatohepatitis (NASH), fibrosis, cirrhosis, and, in a subset of patients, to hepatocellular carcinoma (HCC) [12,13]. HCC—the predominant form of primary liver cancer—accounts for the majority of liver cancer mortality worldwide and presents a major therapeutic challenge due to its aggressiveness, high recurrence rate, and resistance to conventional therapies [14]. In the clinical continuum from NAFLD to HCC, quercetin is a candidate both for disease prevention in early metabolic liver disease and for supporting anticancer intervention.

Mechanistic studies indicates that quercetin exerts multi-targeted effects in HCC models: it induces cell-cycle arrest at multiple checkpoints (G0/G1, S, G2/M), suppresses proliferative and invasive signaling (PI3K/Akt/mTOR, MAPK/ERK, NF-κB, JAK2/STAT3), activates the intrinsic (mitochondria-dependent) apoptotic cascade through downregulation of Bcl-2 family members, and modulates autophagy—where excessive autophagic activation can culminate in programmed cell death [15,16,17,18,19]. Moreover, in vitro and in vivo evidence demonstrates tumor growth inhibition by quercetin in experimental HCC models. Despite this preclinical efficacy, translation is hampered by quercetin’s low oral bioavailability and its extensive metabolic conversion in vivo. The observed biological effects of quercetin may be mediated largely by metabolites rather than the parent compound [20].

In this review, we summarize current knowledge of the molecular mechanisms underlying the protective and anticancer effects of quercetin and its metabolites in NAFLD and HCC. Understanding these molecular mechanisms is essential for the rational development of quercetin- or metabolite-based derivatives in disease prevention and therapy, as well as for advancing knowledge at the molecular level of radical-scavenging mechanisms [21,22,23].

This review employed a systematic retrieval strategy to collect the relevant literature. The literature search primarily covered the following databases: PubMed and Web of Science. The search period was the past 10 years. The types of literature included were original research, reviews, and case reports, and the language of the literature was limited to English. In addition, relevant journals and reference lists were manually searched to further supplement any important literature that might have been missed, and for defining descriptions, this could be traced back to even earlier times.

## 2. Sources of Quercetin and Its In Vivo Metabolic Pathways and Products

**Figure 1:**
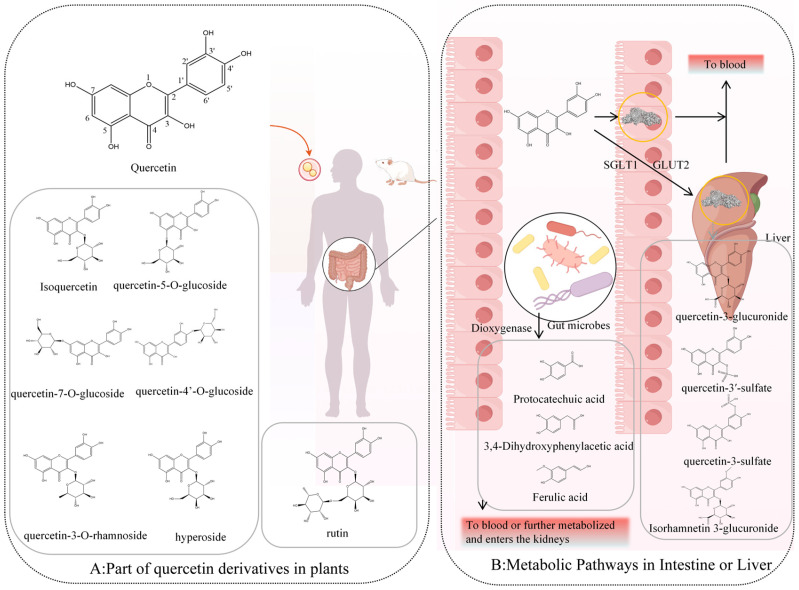
In vivo metabolic pathways and products of quercetin. (**A**): part of quercetin derivatives in plants, (**B**): Metabolic pathways in intestine or liver.

Quercetin predominantly occurs in natural foods and traditional Chinese medicines in glycosylated forms [20] (Figure 1A). In contrast, free quercetin (aglycone) is mainly obtained through dietary supplements rather than whole foods.

Among dietary sources, quercetin-3-O-glucoside (isoquercetin) and its glucuronide derivative, quercetin-3-O-glucuronide, are among the most common forms, found, for example, in lettuce [24]. Quercetin also forms glycosides with other monosaccharides, such as rhamnose and galactose, producing quercetin-3-O-rhamnoside and quercetin-3-O-galactoside (hyperoside). In onions and shallots, quercetin-4′-O-β-glucoside (Q4′G, spiraeoside) is abundant [25], whereas quercetin-3-O-β-rutinoside (rutin)—comprising quercetin linked to the disaccharide rutinose (rhamnose and glucose)—is another dominant form [26]. In traditional Chinese medicines, such as Dianthus superbus, quercetin-7-O-glucoside (quercimeritrin) has been identified [27]. Rare structures, such as quercetin-5-O-glucoside, have been reported in intestinal metabolites of silkworms, though they are uncommon in plants [28]. In the products of some plants, particularly green and fermented teas, flavonoids like catechins become concentrated during processing and aging, resulting in pronounced biological activity when consumed [21].

These glycosylated derivatives are more water-soluble than the aglycone, quercetin. Therefore, glycosylated derivatives are the principal quercetin containing compounds of aqueous and alcoholic herbal extracts, and presumably important contributors to pharmacological activity of these extracts. Because most in vitro and in vivo studies on quercetin bioactivity focus on the aglycone, the following discussion will address primarily the in vivo metabolism of quercetin aglycone.

Intestinal microbiota play a central role in quercetin metabolism and transformation (Figure 1B). Early in vitro fermentation studies with mouse gut microbiota identified 3,4-dihydroxybenzoic acid (protocatechuic acid) and 3,4-dihydroxyphenylacetic acid as primary degradation products, formed via quercetin dioxygenase activity from fungi and bacteria where 2 μmol of quercetin fermented to produce 63 ± 5.9 pM and 4.7 ± 3.1 of protocatechuic acid and 3,4-dihydroxyphenylacetic acid after 24 h, respectively [29]. These compounds are further metabolized into benzoic and phenylacetic acid derivatives [29]. Compared with quercetin itself, such small phenolic acids are more readily absorbed in the colon and achieve higher systemic concentrations.

Beyond microbial degradation, quercetin glycosides can be transported across intestinal epithelial cells through sodium-dependent glucose transporter 1 (SGLT1) and glucose transporter 2 (GLUT2), facilitating entry into enterocytes and hepatocytes [30,31]. Once internalized, phase II enzymes convert them into O-glucuronides and O-sulfates, such as quercetin-3-O-glucuronide and quercetin-3′-sulfate [32]. Methylated derivatives, including isorhamnetin-3-glucuronide, also arise and circulate systemically. In addition, conjugated small-molecule metabolites such as 3,4-dihydroxybenzoic acid sulfate and trans-4-hydroxy-3-methoxycinnamic acid (ferulic acid) have been detected in blood [33].

## 3. Mechanisms Insights

### 3.1. Antioxidant and Redox Modulation

The anticancer effects of quercetin and its metabolites are generally ascribed to their ability to regulate oxidative stress and redox balance. Reactive oxygen species (ROS)—including hydroxyl radicals (OH·), superoxide anions (O_2_·^−^), and hydrogen peroxide (H_2_O_2_)—arise from aerobic metabolism, environmental stressors, or impaired antioxidant defenses [34]. Under physiological conditions, ROS act as signaling molecules, tightly regulated by endogenous antioxidants (e.g., melatonin, uric acid, glutathione), dietary compounds (e.g., vitamins C and E, polyphenols), and enzymatic defenses such as superoxide dismutase (SOD), catalase (CAT), glutathione peroxidase (GPX), and nuclear factor erythroid 2-related factor 2 (NRF2) [35,36].

**Figure 2:**
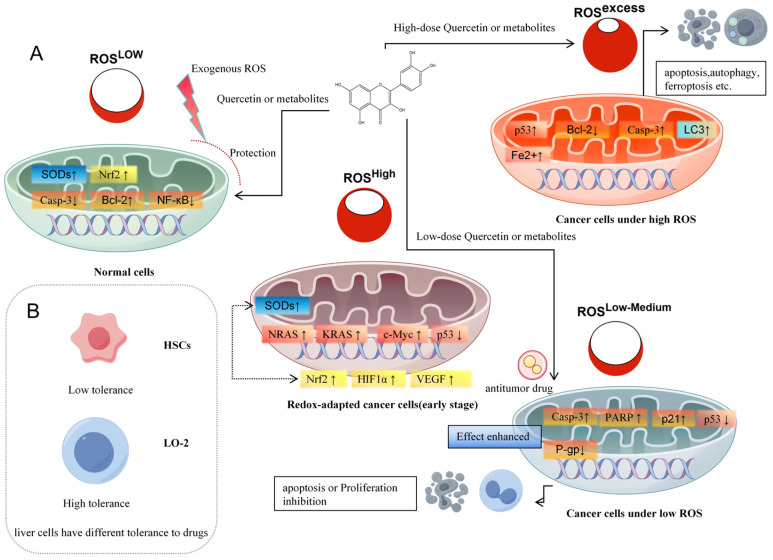
Quercetin and its metabolites modulate liver cancer progression by regulating ROS levels. (**A**): Quercetin responds to different oxidative stress, (**B**): liver cells have different tolerance to drugs.

In tumors, redox homeostasis is frequently disrupted. Oncogenic signaling enhances mitochondrial activity and elevates basal ROS levels relative to normal cells [37] (Figure 2A). In early tumorigenesis, increased ROS contribute to genomic instability and activate mitogenic pathways such as NF-κB, thereby promoting proliferation. Although cancer cells tolerate higher ROS levels than normal cells, excessive ROS levels triggers programmed cell death mechanisms in cancer cells, such as apoptosis, autophagy, and necroptosis [38].

Exploiting ROS imbalance in anticancer therapy has proven clinical effectiveness, evidenced by the effectiveness of radiation therapies. Also the molecular mechanism of some anticancer drug treatments involves excessive ROS formation. For instance, sorafenib—a multikinase inhibitor and FDA-approved first-line therapy for hepatocellular carcinoma (HCC)—induces ROS overproduction in vitro and in vivo in cancer cells, leading to ferroptosis, a regulated cell death pathway characterized by inhibition of the cystine/glutamate antiporter and accumulation of mitochondrial ROS [39]. Current anticancer strategies that target redox regulation generally follow two complementary approaches: (i) elevating ROS beyond the tolerance limit of cancer cells to induce cell death, or (ii) suppressing ROS to inhibit proliferation, invasion, and metastasis as well as to protect “healthy” tissue [38].

Quercetin and its metabolites act as redox modulators, exhibiting both antioxidant and pro-oxidant properties in a concentration- and context-dependent manner. At low concentrations (typically ≤10 µM in vitro), quercetin acts primarily as an antioxidant, scavenging reactive oxygen species (ROS) and chelating transition metals. These effects activate the Nrf2–ARE pathway and upregulate endogenous antioxidant enzymes (e.g., HO-1, NQO1, GCLM), thereby protecting hepatocytes from oxidative stress and preventing malignant transformation [40,41]. In contrast, at higher concentrations (≥30–50 µM) or under conditions of enhanced ROS generation, such as in hepatocellular carcinoma cells, quercetin and certain metabolites shift toward pro-oxidant behavior, for example, by forming reactive semiquinones and quinones, producing ROS, and disrupting mitochondrial function. This induces apoptosis via cytochrome c release, caspase-3/9 activation, and suppression of survival pathways such as PI3K/Akt/mTOR and NF-κB [15,42]. This dual redox role is central to the therapeutic relevance of quercetin—contributing to cytoprotection in normal hepatocytes while enabling selective cytotoxicity in malignant ones.

Their diversity in chemical structure implies that various molecular mechanisms are involved, and caution is needed to put quercetin and its metabolites under the same umbrella. This chemical diversity of the metabolites formed also indicates that the effect of quercetin administration is multifaceted.

#### 3.1.1. Quercetin

Initially the therapeutic effect of quercetin was solely ascribed to its ability to scavenge reactive oxygen or nitrogen species (ROS), thus decreasing the direct cancer promoting effects of ROS. It has been argued that maximum concentration of quercetin that can be reached in vivo is too low to scavenge a physiological relevant quantity of ROS. It should be noted that these quercetin and other polyphenolic compounds—once oxidized—can be regenerated by endogenous compounds in the redox network of the cell, such as ascorbate and glutathione, and thus might function as catalysts in ROS decomposition. Due to the interaction with endogenous compounds, a relatively low concentration might still have a substantial ROS scavenging impact through such interaction [43,44], or else as a fundamental structural aspect of these systems as well as their analogues and metabolites [21,45].

There is consensus that activation of the antioxidative KEAP1-NRF2 pathway is involved in the protective effect of the quercetin and its metabolites. It is supposed that the compounds interact with KEAP1, disrupting the KEAP1-NRF2 interaction, which promotes NRF2 nuclear translocation thus inducing antioxidant enzymes. Several in vitro studies confirm quercetin-induced activation of the NRF2 pathway.

For the molecular mechanism involved in activation of the KEAP1-NRF2 pathway several hypothesis exist. At high concentrations, quercetin and its metabolites generate more ROS. One of the responses to elevated ROS production is activation of the KEAP1-NRF2 pathway. By this feedback mechanism the redox equilibrium of the cell can be kept within narrow limits when ROS levels rise. Alternatively, quercetin and its metabolites might directly interact with KEAP1, disrupting KEAP1-NRF2 interactions and promoting NRF2 nuclear translocation thus inducing antioxidant enzymes. This is substantiated by computational simulations as well as molecular docking [46].

**Figure 3:**
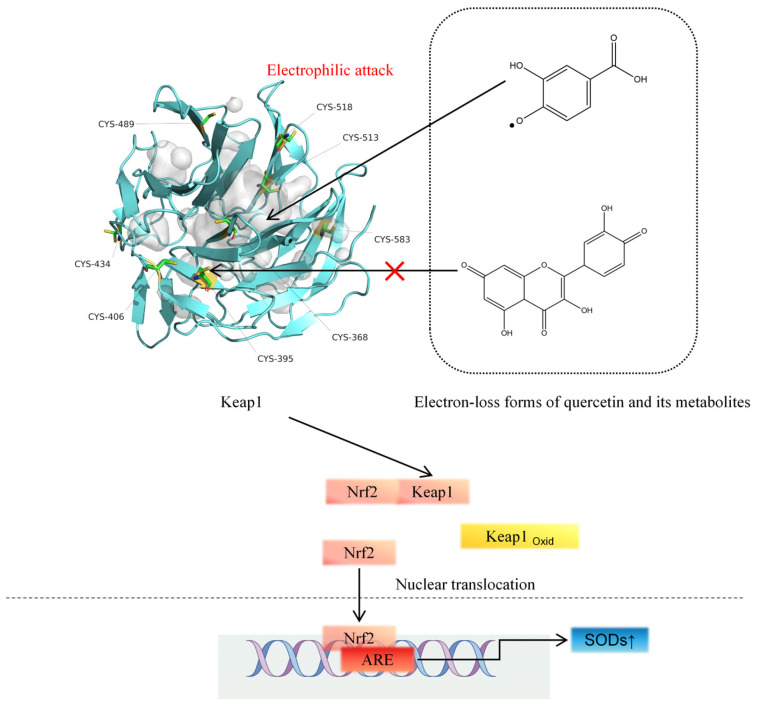
The electron-loss forms of quercetin metabolites electrophilically attack the -SH of the KEAP1 protein.

When quercetin or its metabolites scavenge ROS, this would lead to less activation of the KEAP1-NRF2 pathway by ROS. It should be noted that during ROS scavenging, quercetin will be oxidized to a quercetin semi-quinone radical and quercetin quinone (Figure 3). It has been hypothesized that the oxidative products formed in their chemical reaction with ROS also contribute to the cytoprotective effects of quercetin and its metabolites.

Quercetin quinone is a potent electrophile, and from a chemical point of view, it is prone to covalently bind to, e.g., one of the nucleophilic thiols in KEAP1, thus activating the NRF2 pathway. For nucleophiles such as acrolein, the formation of covalent adducts with thiol residues on KEAP1 has been demonstrated, which has been associated with the activation of the NRF2 pathway by this nucleophile [47,48]. However, a quercetin quinone–KEAP1 adduct could not be detected by Q-TOF in the test tube when quercetin quinone was formed in the presence of KEAP1. For the semisynthetic flavonoid monoHER (7monohydroxeethyl-rutoside), it was found that monoHER quinone forms a monoHER–KEAP1 adduct on a cysteine residue of KEAP1 in vitro [49]. This indicates that the role of the quinones formed in the chemical oxidation of redox modulators like quercetin with ROS warrants further research.

Quercetin quinone is spontaneously converted into 2-(3,4-dihydroxybenzoyl)-2,4,6-trihydroxy-3(2H)-benzofuranone (BZF), a relatively stable oxidation product. BZF has been shown to exert potent radical scavenging and metal-chelating activity at nanomolar concentrations [50,51]. Mechanistically, BZF modulates intracellular signaling by inhibiting NF-κB activation, suppressing inflammatory cytokine expression, and preserving intestinal barrier integrity in models of oxidative stress [52]. Furthermore, BZF has been reported to attenuate ROS-mediated damage by regulating NADPH oxidase activity and activating Nrf2-driven cytoprotective genes, thereby linking it to redox-sensitive transcriptional control [53]. Thus, BZF may also represent an important mediator of quercetin’s health benefits.

Interestingly, since oxidized products of quercetin and other polyphenols are preferentially formed at sites of elevated ROS, quinones and BZF may be naturally “targeted” to locations where they are have to display their biological effect. This site-specific formation may be a crucial mechanism underlying their therapeutic potential.

#### 3.1.2. Quercetin-3-O-Glucuronide

Quercetin-3-O-glucuronide (Q3GA) is the major circulating metabolite of quercetin after oral intake. In general, gluceronides are readily excreted by the kidney, but binding of Q3GA to plasma albumin extends its half-life [54]. Q3GA displays a potent antioxidant activity. In the HepG2 cell injury model, Q3GA increases the activity of antioxidant enzymes such as catalase (CAT), superoxide dismutase (SOD), and reduced glutathione (GSH), while lowering oxidative stress markers including malondialdehyde (MDA) and nitric oxide (NO·). These effects stabilize the intracellular redox balance and help to prevent progression to a more severe liver disease [55].

Wang et al. [56] showed that in adipocytes, Q3GA inhibits mitophagy via the p38–PINK1–PARKIN pathway and mimics NRF2 signaling, thereby inducing browning and mitochondrial thermogenesis, ultimately ameliorating steatosis. These findings suggest a potential role for Q3GA in preventing the development of nonalcoholic fatty liver disease (NAFLD).

Evidence for Q3GA’s role in hepatocellular carcinoma (HCC) remains scarce, so its potential in HCC is extrapolated from other disease models. Choi et al. [57] demonstrated that Q3GA suppresses Th2-mediated immune responses, which are known to promote tumor growth and metastasis. Mechanistically, Q3GA activates the Raf–ERK–Nrf2 pathway in CD4^+^ T cells, leading to ROS overproduction and induction of heme oxygenase-1 (HO-1). HO-1-derived carbon monoxide inhibits CD4^+^ T cell proliferation by reducing IL-2 production, thereby dampening inflammatory cytokine expression (IL-2, IL-4, IL-5, IL-12, IL-13, IFN-γ).

Quercetin-3-glucuronide (Q3GA) accumulates in macrophages within human atherosclerotic lesions, indicating its capacity to modulate macrophage function [58]. Under inflammatory conditions, surface β-glucuronidase released by activated macrophages can deconjugate Q3GA to quercetin [59,60]. Because of its higher lipophilicity, quercetin is preferentially retained at these sites. Moreover, quercetin acts as a more potent redox modulator than Q3GA, since in Q3GA the free hydroxyl group at the 3-position—critical for the redox activity of quercetin—is blocked [61,62]. Consequently, the site-specific deconjugation of Q3GA effectively results in targeted delivery of quercetin to inflamed tissues.

Interestingly, Q3GA has been reported to upregulate peroxisome proliferator-activated receptor γ (PPARγ), a central regulator of NRF2 signaling. While Q3GA itself does not directly bind PPARγ, quercetin does [62], which is consistent with localized quercetin release from Q3GA and its function as a targeted delivery system.

**Figure 4:**
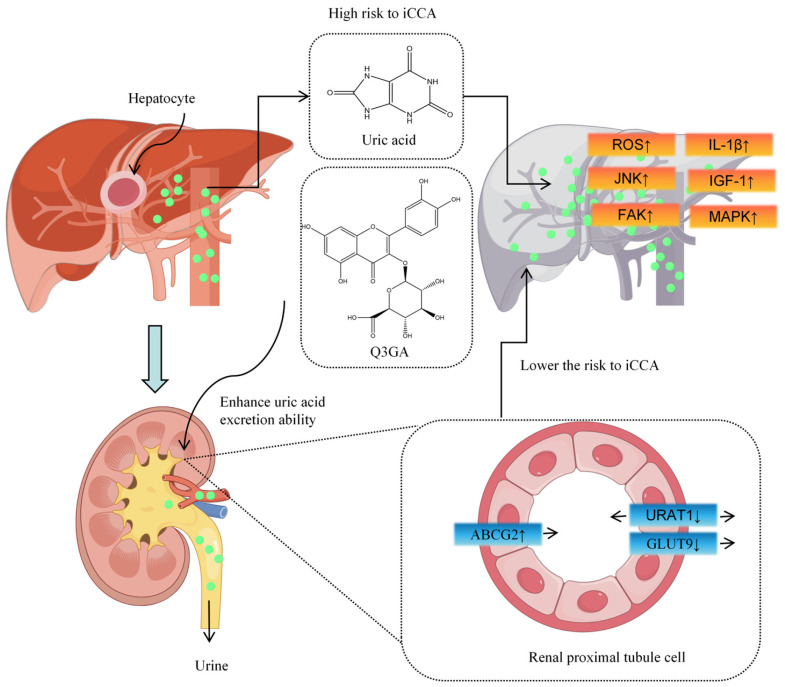
Q3GA reduces liver cancer risk by enhancing uric acid excretion.

Recent Mendelian randomization studies by our group established a causal relationship between uric acid (UA) levels and liver-related diseases, identifying ABCG2-mediated UA elevation as a risk factor for intrahepatic cholangiocarcinoma [63] (Figure 4). Similarly to quercetin, Q3GA significantly inhibits URAT1 and GLUT9 expression while inducing ABCG2 expression in HK-2 cells, reducing UA reabsorption and excretion, thereby lowering UA levels [64]. This suggests a protective role for Q3GA in HCC.

#### 3.1.3. Quercetin-3′-Sulfate and Quercetin-3-Sulfate

Quercetin-3′-sulfate (Q3′S), a major circulating metabolite of quercetin, has a sulfate group at the 3′ position [65]. Studies demonstrate that Q3′S mitigates endothelial-derived nitric oxide (NO.) dysfunction under high oxidative stress induced by the superoxide dismutase (SOD) inhibitor DETCA(copper chelator) and suppresses NADPH oxidase-derived superoxide (O_2_·^−^) release in aortic cells [66]. Similarly to quercetin, Q3′S induces ROS-dependent apoptosis in MCF-7 breast cancer cells [67], though its efficacy in HCC cells requires further investigation. Q3′S also potently inhibits xanthine oxidase (XO), a molybdenum-containing enzyme critical for purine catabolism and produces superoxide (O_2_·^−^). XO inhibition also reduces uric acid formation, potentially lowering HCC risk, and decreases the conversion of the anti-tumor drug 6-mercaptopurine (6-MP) to its inactive metabolite 6-thiouric acid (6-TU), thereby extending the drug’s efficacy [68].

Conversely, quercetin-3-sulfate (Q3S), with a sulfate group at the 3 position, exhibits weaker XO inhibition. In adipocytes, Q3S reduces glucose uptake and triglyceride (TG) assembly, demonstrating lipid-lowering effects [69]. Regarding apoptosis, Q3S upregulates p53 (trp53) but increases anti-apoptotic Bcl-2 and decreases caspase-3, indicating no apoptosis activation [70]. The differential effects of these quercetin metabolites, determined solely by a different position of the sulfate group on the molecule, diversify the biological responses induced by quercetin administration.

#### 3.1.4. Isorhamnetin-3-Glucuronide and Isorhamnetin

Isorhamnetin-3-glucuronide, formed by conjugation of isorhamnetin (a 3′-methylated quercetin derivative) with glucuronic acid via a β-glycosidic bond, is a major circulating form of quercetin, though its physiological activities are understudied [71]. Munkhzul et al. [72] reported that isorhamnetin reduces steatosis and fibrosis in non-alcoholic steatohepatitis (NASH) mouse models by downregulating inflammatory factors, such as TGF-β, in hepatic stellate cells (HSCs) and inhibiting macrophage infiltration, thereby preventing HSC activation. This anti-fibrotic effect may help to prevent NAFLD progression to HCC.

At high doses (>100 μM), isorhamnetin elevates intracellular ROS levels in Hep3B HCC cells, disrupting mitochondrial metabolism, increasing the Bax/Bcl-2 ratio, and releasing cytochrome c, leading to G2/M cell cycle arrest. This effect is reversed by the ROS inhibitor N-acetylcysteine (NAC) [73]. In contrast, low-dose isorhamnetin (25 μM) activates AMP-activated protein kinase (AMPK) in HepG2 cells, protecting against mitochondrial damage from severe oxidative stress [74]. This dual role—promoting ROS at high doses and mitigating ROS damage at low doses—underscores the need for accurate dosing strategies for flavonoids. Research also found that in MHCC-97H xenograft models, isorhamnetin suppresses HCC via the GSK-3β/PI3K/AKT pathway [75].

**Figure 5:**
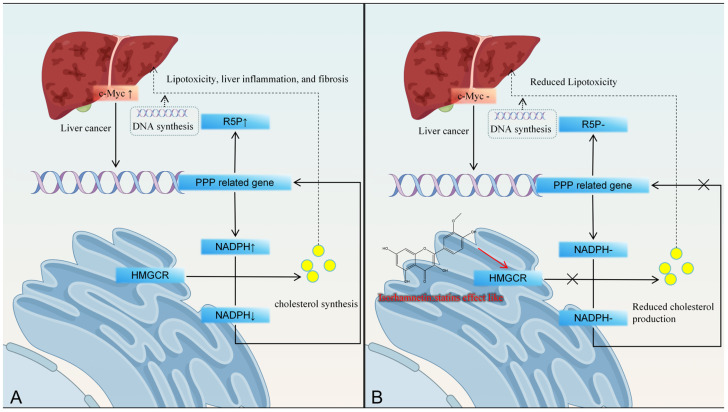
Isorhamnetin inhibits liver cancer progression by reducing cholesterol production through inhibiting HMGCR activity. (**A**): HMGCR affects hepatic lipid metabolism pathways, (**B**): Isorhamnetin inhibits HMGCR activity.

Metabolic reprogramming is a hallmark of cancer, with dysregulated cholesterol synthesis in HCC marked by upregulated HMG-CoA reductase (HMGCR), a rate-limiting enzyme. Isorhamnetin mimics statins by reducing HMGCR activity in HepG2 cells, inhibiting cancer cell proliferation [76,77]. Recent studies suggest that the continuous synthesis of cholesterol contributes to HCC progression. In HCC, activation of the proto-oncogene c-Myc leads to activation of PPP-related genes, which in turn stimulates NADPH synthesis and leads to upregulation of R5P expression. This protein is a substrate for DNA synthesis and promotes HCC cell replication. Cholesterol synthesis continuously consumes NADPH, which in turn stimulates the expression of PPP-related genes and the continuous production of NADPH through positive feedback [78]. Accumulated cholesterol can lead to lipid oxidative toxicity, inflammation, and other factors, promoting HCC progression (Figure 5).

Stem cell differentiation is a potential strategy for repairing damaged livers, with stem cell transplantation being explored for genetic liver diseases or bioartificial liver support systems [79]. Hiroko Isoda et al. [80] found that isorhamnetin induces hepatocyte-specific differentiation of human amniotic epithelial cells (hAECs), upregulating liver progenitor markers EPCAM and DLK1 while downregulating AFP, without evidence of transdifferentiation. However, isorhamnetin does not promote further stem cell maturation. This early differentiation induction may enhance chemotherapy sensitivity, as seen with oncostatin M, which induces EpCAM-positive HCC stem cell differentiation, driving G0-phase cells into the cell cycle and increasing sensitivity to 5-fluorouracil [81]. Whether isorhamnetin exerts similar effects requires further investigation.

#### 3.1.5. 3,4-Dihydroxyphenylacetic Acid

3,4-Dihydroxyphenylacetic acid (DOPAC), a primary degradation metabolite of quercetin produced by gut microbiota, exhibits dual effects: promoting ROS generation at high concentrations and conferring protection at low concentrations [82,83]. Nunes et al. reported that in neuronal cells at concentrations exceeding 200 nmol/10^6^ cells, DOPAC, combined with the NO. donor S-nitroso-N-acetylpenicillamine (SNAP), induces early mitochondrial membrane potential dissipation and ATP depletion, leading to loss of membrane integrity. This cell death is independent of apoptosis, as caspase-3 and caspase-9 remain inactive [84]. At low concentrations, DOPAC inhibits NO. donor-induced canonical apoptosis pathways. In colonic epithelial cells, DOPAC prevents heme-induced ROS enhancement and cytochrome c release, displaying greater activity than quercetin itself [85]. In HCC, DOPAC induces aldehyde dehydrogenase (ALDH) activity, protects against acetaldehyde-induced cytotoxicity, and activates the NRF2 pathway, effects validated in vitro [86].

#### 3.1.6. Protocatechuic Acid

Protocatechuic acid (PCA), another major degradation product of quercetin, has a phenolic hydroxyl and α,β-unsaturated carbonyl groups, conferring both antioxidant and pro-oxidant properties. Like quercetin and isorhamnetin, PCA directly scavenges free radicals and, in normal cells, enhances endogenous antioxidant enzyme activity, including glutathione peroxidase (GSH-PX) and superoxide dismutase (SOD). It reduces xanthine oxidase (XOD), NADPH oxidase (NOX), and malondialdehyde (MDA) levels, while decreasing apoptosis markers caspase-3, annexin-V, and BAX [87,88]. Abdelrahman et al. [89] demonstrated PCA’s protective role against cisplatin-induced hepatic apoptosis. This ROS-suppressive effect also mitigates H_2_O_2_-induced endoplasmic reticulum (ER) stress in HepG2 cells by downregulating genes such as activating transcription factor 4 (ATF4), inositol-requiring enzyme 1α (IRE1α), and phosphorylated p38 MAPK.

In cancer cells, PCA downregulates cyclin D1 to inhibit proliferation, blocking G1-to-S phase transition and elevating p53 to induce apoptosis, effects linked to c-Jun N-terminal kinase (JNK) and p38 activation [90]. Yip et al. [91] reported that 100 μmol/L PCA induces JNK-dependent HCC cell death. JNK1 regulates cyclin D expression via p21 downregulation, c-Myc upregulation, and c-Jun suppression, promoting HCC proliferation. However, excessive ROS activates apoptosis signal-regulating kinase 1 (ASK1) in the JNK pathway, forming an ASK1-MEKK4/7-JNK-ROS positive feedback loop, leading to sustained ROS accumulation, mitochondrial damage, and apoptosis [92,93].

#### 3.1.7. Trans-4-Hydroxy-3-Methoxycinnamic Acid (Ferulic Acid)

Ferulic acid (FA) exerts effects in HCC similar to PCA, promoting ROS generation and activating mitochondria-mediated apoptosis in HepG2 cells [94]. FA treatment enhances LC3-I to LC3-II conversion and autophagosome accumulation, boosting autophagy and inhibiting cancer cell proliferation [95]. Molecular docking suggests FA’s autophagy enhancement may involve targeting signal transducer and activator of transcription 3 (STAT3), mitogen-activated protein kinase 1 (MAPK1), and phosphoinositide-3-kinase regulatory subunit 1 (PIK3R1) [96]. FA also downregulates matrix metalloproteinases (MMPs), including MMP-1, MMP-2, MMP-3, MMP-9, and MMP-12, suppressing tumor cell migration and metastasis, and induces cell cycle arrest at G0/G1 or S phases. In vitro, FA inhibits hypoxia-inducible factor-1α (HIF-1α) expression, mitigating HCC-induced hypoxia and angiogenesis via ERK1/2 and JNK-1 suppression, limiting nutrient supply to cancer cells [97].

At low concentrations, FA, like other metabolites, upregulates antioxidant enzymes (SOD, GPx, CAT), enhancing cellular defense against ROS-induced damage. It suppresses pro-inflammatory mediators, including nuclear factor-κB (NF-κB), cyclooxygenase-2 (COX-2), TNF-α, IL-1β, and IL-6, which are overexpressed in HCC, improving the tumor microenvironment’s oxidative and inflammatory state [98,99]. FA also acts as a direct P-glycoprotein (P-gp) inhibitor in KBChR 8-5 cells, reducing its effective concentration and ABCB1 expression, enhancing chemotherapy sensitivity [100]. However, this effect in HCC requires further validation.

### 3.2. Modulation of the Gut Microbiome

**Figure 6:**
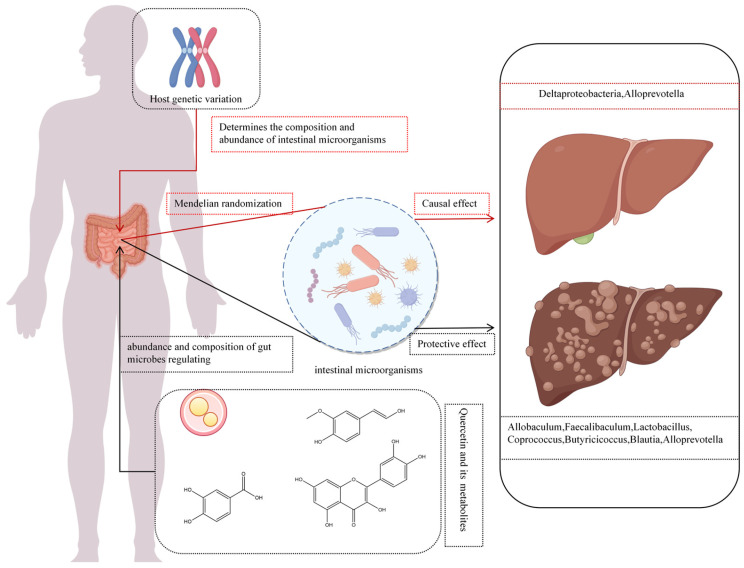
Quercetin and its metabolites affect liver cancer progression through intestinal microbiota.

A novel aspect in the therapeutic effects of quercetin and its metabolites is modulation of the gut microbiome (Figure 6). Gut microbiota influence HCC progression via metabolites that mediate choline metabolism, TLR4 activation, toxin production, and short-chain fatty acid (SCFA) regulation [101,102]. SCFAs—mainly acetate, propionate, and butyrate—play pivotal roles in alleviating hepatic steatosis. Butyrate, for instance, suppresses hepatic lipid synthesis by downregulating the expression of sterol regulatory element-binding protein-1 (SREBP-1) [103], while SCFAs also promote GLP-1 secretion, enhancing hepatic lipid oxidation [104]. However, the effect of acetate may vary with dietary context: Zhang et al. reported that acetate promotes lipogenesis under a high-fructose diet but inhibits NAFLD progression under a high-fat diet [105]. In liver disease, quercetin and its metabolites balance gut microbial communities, enhance intestinal barrier function, and regulate bile acid metabolism to mitigate NAFLD progression. Quercetin supplementation reduces the Firmicutes/Bacteroidetes (F/B) ratio and Erysipelotrichaceae abundance while increasing Proteobacteria abundance [106]. Furthermore, an elevated Firmicutes/Bacteroidetes (F/B) ratio is commonly observed in obesity and NAFLD models [107]. A higher F/B ratio typically reflects enhanced energy extraction from the diet, leading to greater fat accumulation and impaired hepatic lipid metabolism. Fecal microbiota transplantation experiments have shown that transferring the gut microbiota of obese donors into germ-free mice induces hepatic triglyceride accumulation [105,108].

Li et al. [109] found that high-dose quercetin increases anti-inflammatory and SCFA-producing bacteria, including Allobaculum, Faecalibaculum, Lactobacillus, Coprococcus, Butyricicoccus, Blautia, Lacnospiraceae NK4A136, Parabacteroides, and Alloprevotella. Observational studies link elevated Firmicutes relative to Bacteroidetes with fat deposition [110], while SCFAs correlate with inflammation. Combined with anti-PD-1 therapy, quercetin enhances Firmicutes and Actinobacteria abundance, upregulating M2 macrophage-related genes (Arg-1, IL-10, TGF-β, MMP-9) and suppressing M1-related genes (IL-6, IL-12a, IL-1β, TNF-α), improving the HCC immune microenvironment [111].

Similarly, ferulic acid inhibits the growth of harmful bacteria like Helicobacter pylori and increases the abundance of SCFA-producing bacteria (*p* < 0.05) [112]. In addition, dietary PCA enhances Bacteroidetes abundance, reducing hepatic cholesterol and lipid accumulation [113,114]. All these effects contribute to its ability to ameliorate NAFLD progression.

### 3.3. Other Mechanisms

Advances in bioinformatics, particularly Mendelian randomization (MR), have validated causal relationships between microbial abundance and liver diseases. MR leverages genetic variants as instrumental variables to mimic randomized controlled trials, mitigating confounding and reverse causation biases in observational studies and elucidating causal chains from genetic variation to molecular phenotypes to disease [98,99]. For instance, Alloprevotella, a Gram-negative obligate anaerobe producing acetate and succinate, exerts protective effects against alcoholic liver disease, liver failure, benign liver tumors, and primary liver malignancies [115]. Zhou et al. [116] identified a protective causal link between genetically driven Deltaproteobacteria abundance and NAFLD risk. These findings also contribute to solving the puzzle on how quercetin and its metabolites modulate liver diseases via the gut microbiome (Figure 6).

## 4. Cell-Specific Effects and Targeting

The effects of quercetin and its metabolites vary widely depending on cell type, concentration, and local microenvironment, complicating evaluation of their therapeutic potential (Figure 2B). For example, quercetin inhibits hepatic stellate cell (HSC) proliferation above 20 μM and induces apoptosis at 40 μM via reduced Bcl-2 expression [117], while normal hepatocytes remain unaffected until 80 μM. Similarly, 60 μM quercetin is non-toxic to human umbilical vein endothelial cells [118] but induces apoptosis in HepG2 cells at ≤40 μM [119]. Metabolites of quercetin show comparable concentration- and cell type-specific responses [75,120]. These differences highlight the need for careful dose-finding strategies.

The difference between “normal” cells and unhealthy cells, including the difference in the local environment of the cell such as pH and oxygen tension, needs more attention. This can be used to “target” the effect of quercetin administration. A nice example is the production of oxidized products of quercetin and other polyphenols (namely quinones and BZF) at sites of elevated ROS where they have to display their desired effect. Another example is the formation of quercetin out of Q3GA at sites of inflammation. The mechanisms that direct the effect quercetin administration to the desired sites of action—by exploiting differences between normal and unhealthy cells—warrant more in-depth investigation.

## 5. Discussion and Future Perspectives

Current research strongly supports that quercetin and its metabolites exert protective, anti-fibrotic, and anti-tumor effects, primarily mediated by redox modulation and microbiota-dependent pathways. These findings highlight quercetin’s significant potential as a dietary and therapeutic agent for the prevention and management of NAFLD and HCC. However, several key challenges must be addressed to realize this potential. These include quercetin’s low oral bioavailability, which is a major hurdle for clinical translation [121,122]. Furthermore, potential redox–drug interactions necessitate careful evaluation before quercetin is combined with targeted cancer therapies [123,124]. Finally, persistent gaps remain in our understanding of the precise molecular mechanisms driving its biological effects.

To overcome these limitations, several promising strategies are being explored. These include the development of nanoformulations and encapsulation systems, such as liposomes and polymeric nanoparticles, to enhance solubility, stability, and bioavailability [125]. Another approach involves creating prodrugs and (semi)synthetic derivatives with improved pharmacokinetic profiles [126]. Additionally, combination therapies that pair quercetin with conventional drugs are being investigated to improve overall efficacy while minimizing adverse interactions [127].

## 6. Conclusions

In our opinion, future research should prioritize elucidating the precise molecular mechanisms underlying the effects of quercetin and its metabolites, as these form the foundation for their rational therapeutic use in liver-related diseases. In particular, mechanisms that direct the effect of these bioactive compounds to their desired sites of action—by exploiting differences between normal and tumor cells—warrant more in-depth investigation.

## Data Availability

No new data were created or analyzed in this study.
